# Assessment of trabecular bone score using updated TBS_TT_ in anorexia nervosa—The AN-BO study

**DOI:** 10.1371/journal.pone.0311499

**Published:** 2024-10-18

**Authors:** Judith Haschka, Martina Behanova, Didier Hans, Annina Arens, Christian Muschitz, Larisa Dzirlo, Julia Binder, Stylianos Kapiotis, Jochen Zwerina, Heinrich Resch, Roland Kocijan

**Affiliations:** 1 1st Medical Department Hanusch Hospital, Ludwig Boltzmann Institute of Osteology at Hanusch Hospital of OEGK and AUVA Trauma Center Meidling, Vienna, Austria; 2 Medical Department II, Karl Landsteiner Institute for Gastroenterology and Rheumatology at Rheuma-Zentrum Wien Oberlaa and St. Vincent Hospital, Vienna, Austria; 3 Interdisciplinary Center of Bone Diseases, Lausanne University Hospital & University of Lausanne, Lausanne, Switzerland; 4 Medical Department II, St. Vincent Hospital, Vienna, Austria; 5 HealthPi Medical Center, Vienna, Austria; 6 Medical Department III, St. Vincent Hospital, Vienna, Austria; 7 Department of Obstetrics and Gynecology, Medical University of Vienna, Vienna, Austria; 8 Labcon—Medical Laboratories, Vienna Austria; Federal University of Minas Gerais: Universidade Federal de Minas Gerais, BRAZIL

## Abstract

**Objective:**

Anorexia Nervosa (AN) is characterized by a distortion of body image, very low body weight, malnutrition and hormonal dysregulations, resulting in reduced bone mineral density (BMD) and impaired bone microarchitecture. The updated Trabecular Bone Score (TBS) algorithm accounts for soft tissue thickness (TBS_TT_) instead of BMI (TBS_BMI_). The aim of the study was to assess both TBS algorithms in adult AN patients compared to normal-weight controls(CTRL).

**Method:**

This retrospective cross-sectional study investigated 34 adult female anorexia nervosa (AN) patients and 26 healthy normal-weighted age- and sex-matched controls (CTRL). Bone texture analysis was assessed by TBS_TT_ and TBS_BMI_ (TBS iNsight^®^ V4.0 and V3.1), bone mineral density (BMD; lumbar spine LS, femoral neck, total hip) and body composition by DXA (GE Lunar iDXA^TM^). Laboratory analyses included bone turnover markers (CTX; P1NP; sclerostin). Data analysis was performed using parametric (t-test) or non-parametric test (Mann-Whitney-U-Test) depending on normality, one-way ANCOVA and correlation analysis (Perason’s or Spearman’s).

**Results:**

AN patients **(**BMI 14.7(1.6)) and CTRL (BMI 22.4(4.0)) were of comparable age (22.8(7.1) vs.25.0(4.0)years, p = 0.145). TBS_TT_(1.319±0.09 vs.1.502±0.07, p<0.001) and TBS_BMI_(1.317±0.10 vs.1.548±0.09, p<0.001) were significantly lower in AN patients compared to CTRL. Soft tissue thickness was lower in AN (p<0.001). Within the CTRL group, but not in AN, TBS_TT_ and TBS_BMI_ were significantly different (p<0.001). BMD was lower at all sites in AN patients (p<0.001 for all), being lowest at LS. Bone Mineral Content, Lean Body mass and Fat Mass were lower in AN (p<0.001). AN patients had lower P1NP (p = 0.05), but higher CTX (p = 0.001) and sclerostin (p = 0.003) levels.

**Conclusion:**

Adult AN patients have lower TBS_TT_ and TBS_BMI_, reduced BMD and an uncoupling of bone turnover. In AN both TBS algorithms show similar reduced trabecular bone microarchitecture. The observed difference of TBS_TT_ and TBS_BMI_ in CTRL with normal body composition highlight the importance of the new algorithm.

## Introduction

Anorexia Nervosa (AN) is a psychiatric disorder associated with low body weight, predominantly affecting females and the disease manifests in up to 75% during adolescence [1]. The overall lifetime prevalence of AN ranges from 0.1–3.6% in females and 0%-0.3% in males [2]. Patients with AN have multiple risk factors, all of them contributing to reduced bone mineral density (BMD) and bone microarchitecture. In addition to malnutrition, low body mass index (BMI), reduced intake of calcium and deficiency of essential compounds of a healthy diet, AN patients have a severe disbalance of hormonal axes, e.g. hypogonadotropic hypogonadism resulting in amenorrhea, growth hormone resistance and hypercortisolemia [3,4]. Despite these essential risk factors, the early disease manifestation in adolescence and young adulthood is further counteracting the gain of peak bone mass [5]. Further, increased bone resorption markers accompanied by decreased bone formation markers have been previously described in adult AN patients, indicating an uncoupling of bone turnover [4]. Furthermore, sclerostin, an important inhibitor of the Wnt/beta-catenin pathway and thus bone formation, showed elevated levels in young adult AN patients [6]. However, BMD only partially determines bone strength [7,8]. Ex vivo studies demonstrated that BMD predicts only around 66–74% of the variation in bone strength [9]. Assessment of areal BMD using 2-dimensional DXA scanning has the ability to predict fracture risk but not to identify individual persons who will sustain a fracture [8]. Fracture risk assessment improves by including known clinical risk factors [10], but additional bone microarchitecture and bone geometry also significantly account to bone strength and consequently fracture risk [9]. Deterioration of cancellous and cortical bone microarchitecture are hallmarks for osteoporosis and deterioration of bone volume and architectural variables assessed by microcomputed tomography explain up to 90% of strength variability [11]. Adolescence is critical for bone accrual since 95% of peak bone mass is acquired by the age of 18 years and trabecular bone morphology changes from rod-like to plate-like trabeculae, which is further associated with bone strength [12,13]. Therefore, AN patients have not only a combination of risk factors for bone loss but also for failing to accrue bone mass and bone microarchitecture.

Trabecular Bone Score (TBS) is a texture parameter based on an experimental variogram of grey levels across 2-dimensional DXA scans and therefore providing an indirect index for trabecular bone microarchitecture [14,15]. A higher TBS value is accompanied with greater trabecular connectivity, while lower levels reflect higher separation between the trabeculae. Low lumbar spine TBS is associated with the history of fracture as well as risk for new fractures, largely independent of BMD [16]. Therefore, TBS is an independent predictor of osteoporotic fractures and adds an additive information in fracture risk prediction apart from BMD and without additional radiation [14,17,18]. However, the presence of increased regional soft tissue leads to alterations in X-ray absorption and may therefore underestimate TBS due to decreased grey level variations [19,20]. The updated TBS software (TBS iNsight®v4.0) provides an updated algorithm accounting for soft tissue thickness (TBS_TT_) instead of body mass index (BMI, TBS_BMI_) to reduce this bias [20]. TBS_BMI_ uses a surrogate tissue thickness for a population with a BMI of 15 to 37kg/m^2^ [21]. However, BMI provides no information on body fat mass or fat mass distribution. This is of special interest in terms of comparison of patient populations with different body compositions.

The aim of the study was to investigate TBS, using both available software applications, the new TBS_TT_ software and TBS_BMI_, in a cohort of adult patients with AN and consequently low BMI, lean body mass and fat mass compared to healthy controls with normal BMI. Secondary objectives included the assessment of BMD, body composition by DXA, established bone turnover markers as well as circulating sclerostin levels in both study groups.

## Patients and methods

### Study population

In this retrospective cross-sectional analysis, adult female patients with anorexia nervosa (AN, age median (IQR) 22.8 (7.1) years) and healthy normal-weighted controls (CTRL, age median (IQR) 25.0 (4.0)) were investigated. AN patients were recruited at the Medical Department III at the St. Vincent hospital Vienna/Austria, a specialized medical department for psychosomatic diseases. All patients were diagnosed according to ICD-10 codes for AN. Inclusion criteria for AN were i) body mass index (BMI) ≤ 17.5 kg/m^2^, ii) age between 18 and 50 years, iii) no previous low-trauma fractures or bone-affecting concomitant medications. CTRLs were female and age-matched, with normal body weight (BMI 18.5–24.9 kg/m^2^) and no history of prevalent fractures or bone-affecting medication. Body height and weight were measured. BMI was calculated by body weight (kg)/body height^2^(m). The study was approved by the local ethics committee of the Vinzenz Group (EK24/2022) and conducted in accordance with the declaration of Helsinki.

### Laboratory analyses

Blood sampling was performed after overnight fasting between 8:00 and 10:00 am and stored at -70°C for later analysis. Established bone turnover marker (CTX, CrossLaps; P1NP, procollagen type 1 amino terminal propeptide of type I procollagen), intact parathyreoid hormone (PTH) and 25-hydroxyvitamin D (25(OH)-vitamin D) were measured via chemiluminescence (IDS-iSYS microparticle immunoassay system, Immunodiagnostics Systems Ltd). Calcium was determined photometrically (Architect ci8200, Abbott Laboratories). Serum Cortisol was determined via chemoluminiscence on Architect ci8200 (Abbott Laboratories), serum sclerostin levels were quantitatively determined using an established enzyme immunoassay kit (Biomedica, intraassay coefficient of variation (CV) 5%-6%, interassay CV 2%-6%). Estradiol, lutein hormone (LH) and follicle stimulating hormone (FSH) were assessed using chemiluminescence immunoassays (Axsym, Abbott Laboratories).

### Bone mineral density and body composition

Bone mineral density (BMD) of the femoral neck, total hip and lumbar spine were assessed using Dual Energy X-ray absorptiometry (DXA, *GE LUNAR iDXA™*, *Lunar Corp*., *Madison*, *WI*, *USA*, *software version Encore 13*.*50*.*040*). The assessment of body composition was performed using whole body scan by Lunar iDXA^TM^. The following parameters were obtained: bone mineral content [BMC (kg)], fat mass [FM (kg)], percentage of bodyfat (%FM), total lean body mass [LBM (kg)] and percentage of lean body mass (%LBM). Additionally, lean body mass index (LBMI = LBM (kg)/height^2^) and fat mass index (FMI = FM (kg)/height^2^) were calculated. According to the ISCD guidelines for females aged younger than 50 years, Z-Scores and BMD were reported and a Z-Score lower of -2.0 is defined as “below the expected range for age” [22]. Cut-off points for FMI and FMI were used according to Austrian reference values on body composition analysis using GE-Healthcare DXA system: females 18–29 years FMI 7.8 ± 3.0 kg/m^2^ and LBMI 14.1 ± 1.5 kg/m^2^ [23]. DXA scans were performed by a well-trained and IOF-ISCD certified technician and daily cross-calibrations with standardized control phantoms were conducted for validation. In vivo precision error of DXA scanning was 0.41% for lumbar spine and 0.53% for total hip (coefficient of variation, CV). All results are presented in absolute BMD values (g/cm^2^) and Z-Scores.

### Trabecular bone score–TBS_TT_ and TBS_BMI_

Trabecular bone score was assessed retrospectively out of available lumbar spine DXA scans using the two available TBS algorithms: TBS_BMI_ accounting for BMI using TBS iNsight^®^ software version 3.1 (Medimaps group, Geneva, Switzerland) and TBS_TT_ accounting for soft tissue thickness using TBS iNsight^®^ software version 4.0 (Medimaps group, Geneva, Switzerland). TBS was calculated as the mean of each individual TBS of each included vertebral body. Soft tissue thickness was assessed using TBS iNsight software version 4.0 (Medimaps group, Geneva, Switzerland).

### Statistical analysis

Characteristics of AN and healthy controls were described using means and standard deviation (SD) or medians and interquartile ranges (IQR), based on a normality distribution. We assessed distribution of each parameter via normality plots and by Shapiro-Wilk test. Variables which were not normally distributed were log-transformed or square-root transformed and tested again for normality. If the transformed variable was normally distributed, we proceeded with parametric test, otherwise with non-parametric. Differences between AN and CTRLS in all parameters were assessed by Independent Samples t-test or by Independent Samples Mann-Whitney U test. Paired samples t-test was used to assess statistical difference between TBS_TT_ and TBS_BMI_ within AN and CTRLS. A One-way ANCOVA was conducted to determine a statistically significant difference between AN and CTRLS on BMD controlling for BMI and on TBS_TT,_ TBS_BMI_ after adjusting for BMD of lumbar spine. For exploration of an association between two continuous variables we calculated either Pearson’s correlation coefficient (r) or Spearman’s rank correlation coefficient (r_s_), according to the normality of variable distribution. Statistical analyses were conducted using IBM^®^ SPSS^®^ Statistics for Windows, version 28 (IBM Corp., Armonk, NY, USA).

## Results

### Demographic characteristics

In total, 60 females with AN (N = 34) and CTRLs (N = 26) of comparable age (22.8 (7.1) vs 25.0 (4.0) years, p = 0.145) were included in this analysis. *Table 1* summarizes demographic and disease-specific characteristics. Median BMI of AN patients was 14.7 (1.6) kg/m^2^ and significantly lower compared to normal-weighted controls. Despite the expected lower body weight (p<0.0001), body height was also lower in AN patients (p<0.02). The disease duration of AN was 5.0 (8.0) years and 73% of patients had secondary amenorrhea with a median duration of 1.0 (3.5) years.

**Table 1:** 

	AN	CTRL	p
	N = 34	N = 26	
*Demographic characteristics*			
Age, years	22.8 (7.1)	25.0 (4.0)	0.145
Height, cm	165 ± 6	169 ± 7	**0.02**
Weight, kg	40 ± 4	67 ± 11	**<0.0001**
BMI, kg/m^2^	14.7 (1.6)	22.4 (4.0)	**<0.0001**
Disease duration, years	5 (8)	0 (0)	n.a.
Amenorrhea, N (%)	24 (73)	0 (0)	n.a.
Amenorrhea duration, years	1 (3.5)	0 (0)	n.a.
*Laboratory Results*			
Calcium, mmol/l	2.3 (0.2)	2.3 (0.1)	0.386
PTH, pg/L	36 ± 17	29 ± 12	0.072
25(OH)vitamin D3, ng/ml	27 ± 11	33 ± 10	**0.03**
P1NP, μg/mL	37 (42)	47 (32)	**0.05**
CTX, ng/L	0.728 (0.482)	0.370 (0.305)	**0.001**
Sclerostin, pmol/L	12.6 (10.0)	8.7 (6.9)	**0.003**
Cortisol, μg/dL	18.7 ± 6.8	15.8 ± 8.3	0.146
Estradiol, pg/mL	21 (27)	51 (60)	**<0.0001**
LH, U/L	0 (1)	4 (7)	**<0.001**
FSH, U/L	2 (5)	4 (5)	0.116
*Bone Mineral Density*			
L1-L4 BMD, g/cm^2^	0.973 ± 0.149	1.289 ± 0.146	**<0.0001**
L1-L4 Z-Score, SD	-1.1 ± 1.1	0.7 ± 1.2	**<0.0001**
Total Hip BMD, g/cm^2^	0.841 ± 0.131	1.096 ± 0.116	**<0.0001**
Total Hip Z-Score, SD	-0.9 ± 1.2	0.7 ± 1.0	**<0.0001**
Femoral Neck BMD, g/cm^2^	0.864 (0.196)	1.083 (0.081)	**<0.0001**
Femoral Neck Z-Score, SD	-0.8 (1.9)	0.7 (0.7)	**0.001**
*Body Composition*			
FM, kg	4.9 (5.2)	21.5 (7.8)	**<0.0001**
%FM	13.1 (12.3)	35.6 (5.6)	**<0.0001**
FMI, kg/m^2^	1.8 (1.6)	7.9 (2.5)	**<0.0001**
LBM, kg	32.6 (5.0)	41.0 (7.6)	**<0.0001**
%LBM	86.9 (12.3)	64.9 (5.3)	**<0.0001**
LBMI, kg/m^2^	12.1 (1.7)	14.8 (1.7)	**<0.0001**
BMC, kg	2.0 ± 0.3	2.6 ± 0.3	**<0.0001**
BMD, g/cm^2^	0.990 ± 0.116	1.198 ± 0.103	**<0.0001**
Z-Score, SD	0.18 ± 1.15	1.06 ± 0.99	**0.007**
*Trabecular Bone Score*			
TBS_TT_	1.319 ± 0.09	1.502 ± 0.07	**<0.0001**
Soft Tissue Thickness, cm^2^	13.8 ± 1.02	18.6 ± 2.07	**<0.0001**
TBS_BMI_	1.317 ± 0.10	1.548 ± 0.10	**<0.0001**

*AN*, *Anorexia Nervosa; CTRL*, *healthy controls; BMI*, *body mass index; PTH*, *intact parathyroid hormone; 25(OH)vitamin D3*, *25-hydroxyvitamin D3; P1NP*, *procollagen type 1 amino terminal propeptide of type I procollagen; CTX*, *CrossLaps; LH*, *lutein hormone; FSH*, *follicle stimulating hormone; BMD*, *bone mineral density; TBS*_*TT*_, *trabecular bone score accounting for tissue thickness; TBS*_*BMI*_, *trabecular bone score accounting for body mass index; FM*, *fat mass; FMI*, *fat mass index; LBM*, *lean body mass; LBMI*, *lean mass index; BMC*, *bone mineral content; n*.*a*.*–not applicable*. *All results presented in mean±SD or median (IQR) if not stated otherwise*. *Level of significance p = 0*.*05*.

### Laboratory results

All laboratory results are summarized in *Table 1*. Mean 25(OH)Vitamin D3 levels were significantly lower in AN patients (ng/ml; 26.7±11.0 vs. 32.9±0.9, p = 0.028), though within normal range. Bone turnover markers showed marked differences between the two groups with higher levels of CTX (0.728 (0.482) vs. 0.370 (0.305), p = 0.001) and lower levels of P1NP (37 (43) vs. 47 (32), p = 0.05) in AN compared to CTRLs. Serum sclerostin levels were significantly higher in AN (12.6 (10.0) vs. 8.7 (6.9), p = 0.003). Hormonal analyses showed significantly downregulated levels of estradiol (p<0.0001) and LH (p<0.001) in patients with AN, with no difference in FSH and cortisol levels.

### Bone mineral density

In AN BMD was significantly decreased at all sites compared to CTRL (p<0.0001 for all). The most pronounced reduction of BMD in AN was at the lumbar spine with a Z-Score of -1.1±1.1 compared to normal levels in healthy CTRL (p<0.0001). There were significant group differences in mean BMD at lumbar spine [F (1,57) = 9.404, p = 0.003] and in mean Z-score of total hip [F (1,47) = 5.664, p = 0.02] whilst adjusting for BMI. BMD results at all sites correlated positively with each other (p<0.0001 for all). Further, BMD at all sites was positively correlated with BMI in the total population (lumbar spine r = 0.711, femoral neck r = 0.642, total hip r = 0.670, p<0.0001 for all). Results of BMD at all sites, TBS_TT_ and TBS_BMI_ and body composition are summarized in *Table 1*.

### Trabecular bone score (TBS_TT_ and TBS_BMI_)

**Fig 1:**
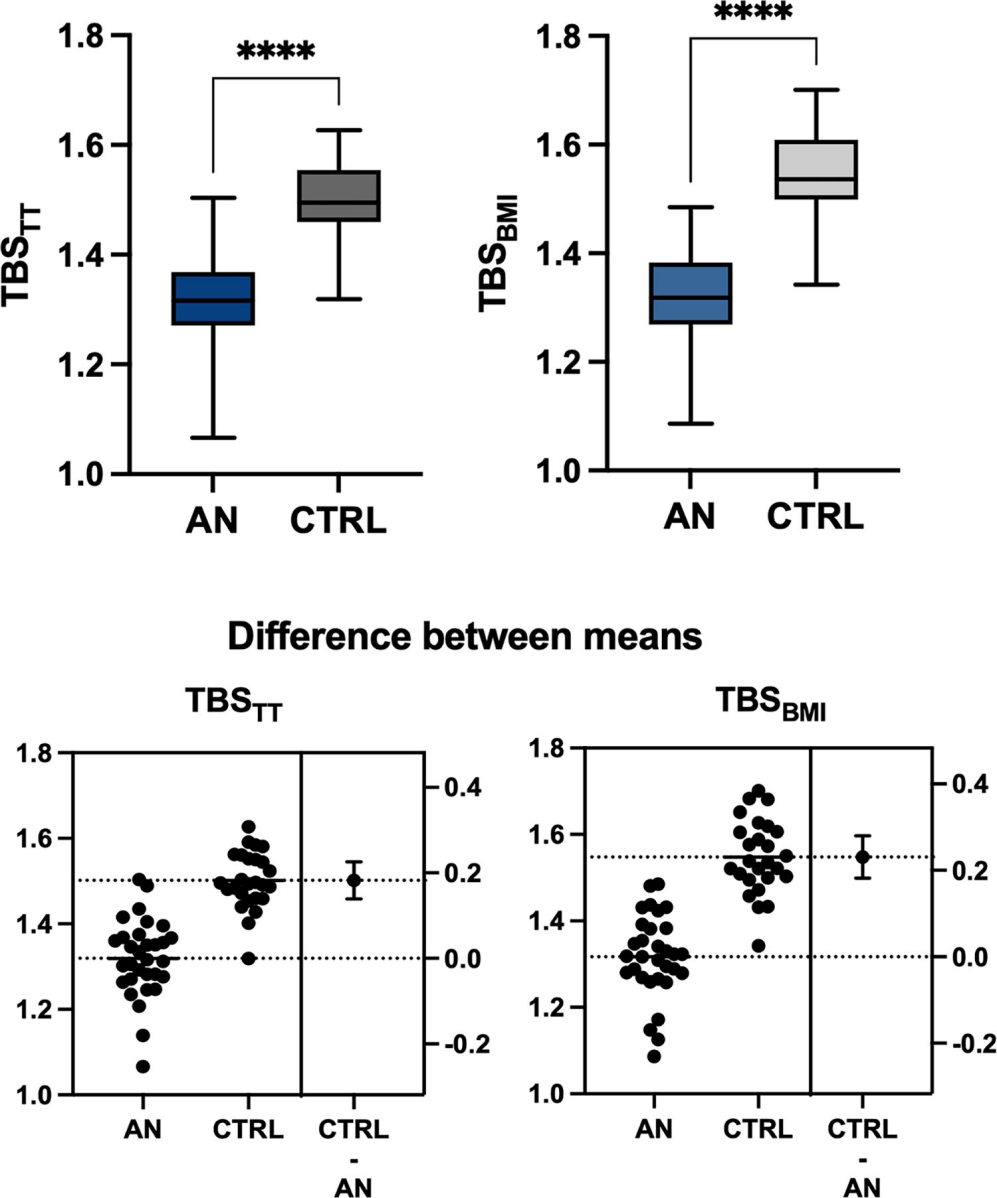
Reduced bone texture parameters, TBS_TT_ and TBS_BMI_, in patients with anorexia nervosa compared to controls. AN, Anorexia Nervosa; CTRL, healthy normal weight controls; TBS_TT_ trabecular bone score accounting for tissue thickness; TBS_BMI_, trabecular bone score accounting for body mass index; Level of significance p = 0.05 (Asterix).

**Fig 2:**
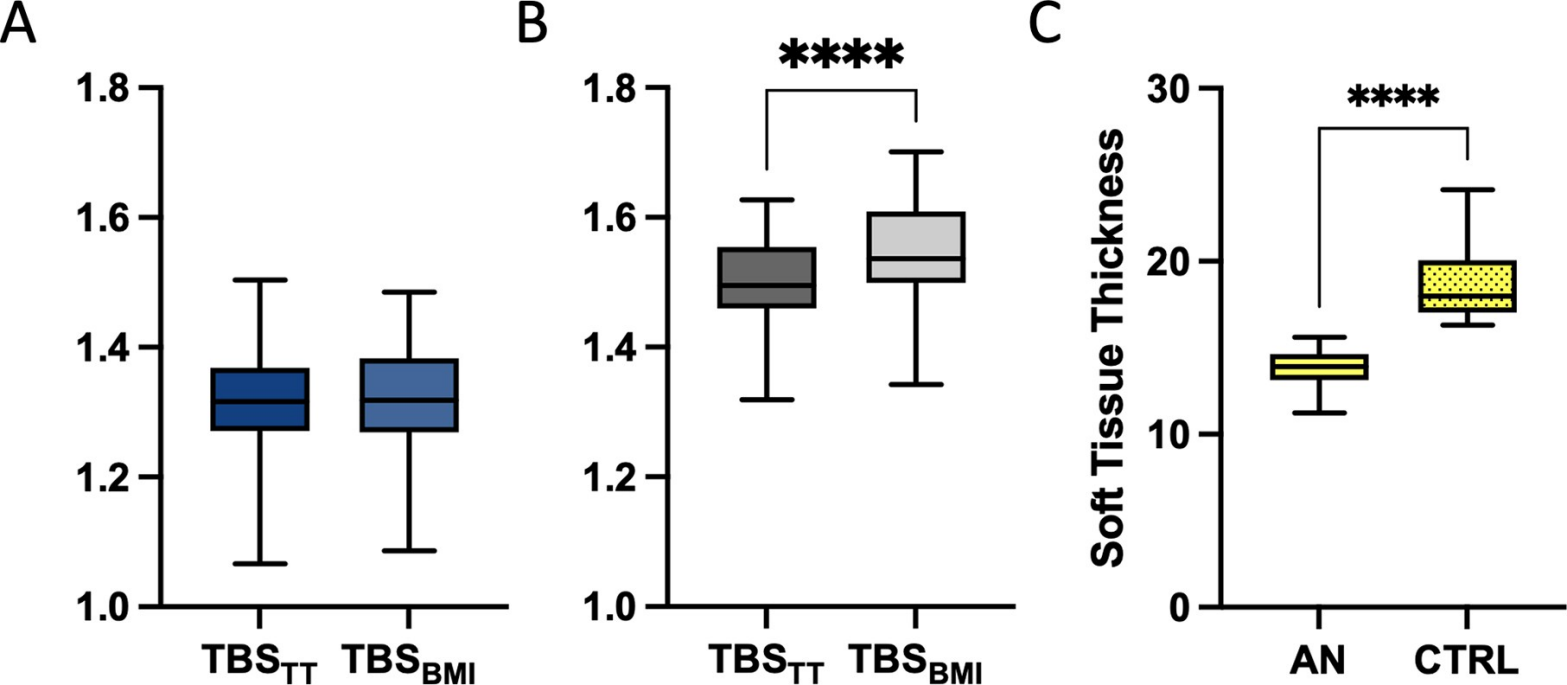
Differences between the two TBS algorithms within anorexia nervosa patients (A) and controls (B) and soft tissue thickness between anorexia nervosa and controls (C). *(A) AN*, *Anorexia Nervosa patients; (B) CTRL*, *healthy normal weight controls; TBS*_*TT*_ *trabecular bone score accounting for tissue thickness; TBS*_*BMI*_, *trabecular bone score accounting for body mass index; (C) Soft tissue thickness in Anorexia Nervosa and controls; Level of significance p = 0*.*05 (Asterix)*.

TBS_TT_ (1.319 ± 0.09 vs. 1.502 ± 0.07, p<0.0001) and TBS_BMI_ (1.317 ± 0.10 vs. 1.548 ± 0.10, p<0.0001) were significantly lower in AN compared to CTRL. (Fig 1) There were significant differences between AN and CTRLS in mean TBS_TT_ [F (1,53) = 6.538, p = 0.013] and in mean TBS_BMI_ [F (1,53) = 12.903, p = 0.001] after adjusting for BMD of lumbar spine. Soft tissue thickness was significantly lower in AN group (cm^2^; 13.8 ± 1.02 vs. 18.6 ± 2.07, p<0.0001). There was no significant difference between TBS_TT_ and TBS_BMI_ within the AN group (p = 0.7), but TBS_TT_ was significantly lower compared to TBS_BMI_ in the CTRL group (p<0.0001). (Fig 2) A strong positive correlation of TBS_TT_ and TBS_BMI_ (r = 0.958, p<0.0001) was observed. Both TBS results correlated positively with BMD of the lumbar spine and hip (r>0.8 and p<0.0001 for all). Duration of amenorrhea correlated negatively with BMD at lumbar spine (r = -0.644, p<0.0001). TBS_TT_ and TBS_BM_ showed weak negative correlations with duration of amenorrhea (r = -0.312 and r = -0.364), reaching level of significance only for TBS_BMI_ (p = 0.05) but not for TBS_TT_ (p = 0.09).

### Body composition

FM, %FM, LBM and BMC were markedly decreased in AN (p<0.0001 for all). In relation to body weight, %LBM was increased in AN patients compared to CTRL (86.9 (12.3) vs. 64.9 (5.3), p<0.0001), but lean body mass index (LBMI) and fat mass index (FMI) were significantly lower in AN patients (p<0.0001 for both). Further, BMD and Z-Score were significantly decreased in AN compared to CTRL (p<0.0001 and p = 0.007, respectively). Positive correlations of BMD of lumbar spine, femoral neck and total hip with LBMI (r = 0.532, r = 0.582 and r = 0.609, p<0.0001 for all) and FMI (r = 0.732, r = 0.621 and r = 0.653, p<0.0001 for all) were observed. No correlation of duration of amenorrhea and body composition was observed. All results of body composition analysis are presented in *Table 1*.

## Discussion

Anorexia nervosa is a complex eating disorder that can manifest itself in impaired bone health, leading to reduced bone mineral density and bone microarchitecture [24–30]. With TBS_TT_ a new algorithm has been introduced, accounting for soft tissue thickness instead of BMI. Since increased soft tissue thickness leads to alterations in X-ray absorption, variations in TBS estimation may occur. Therefore, TBS_TT_ is not only important in patients with increased trunk fat mass, but especially when comparing patient cohorts with different body compositions. In the present study, TBS_TT_ and TBS_BMI_ were significantly decreased in AN patients compared to healthy normal-weight controls. However, while no difference in TBS_TT_ and TBS_BMI_ has been observed in AN, in this normal weight population, a significant difference of TBS_TT_ and TBS_BMI_ has been observed.

A major advantage of TBS is the potential to provide information on trabecular bone microarchitecture from 2-dimensional DXA scans [14,15]. No additional examination, radiation or invasive procedure is needed. An independent prediction of fracture risk by TBS apart from BMD has been previously demonstrated [14,17,18]. Obesity leads to attenuation of X-ray absorption with a direct influence not only on BMD measurement and increase on radiation dose, but further leading to a potential technical limitation of TBS [19,31]. TBS_BMI_ applies a surrogate tissue thickness for a population with BMI of 15 to 37kg/m^2^ [21]. Therefore, in patients with a BMI outlining this range, TBS_BMI_ should not be applied in clinical routine. However, despite that, BMI gives no information about distribution of soft tissue thickness and body fat distribution. The new algorithm uses a built-in correction for soft tissue thickness provided by the densitometer and therefore overcoming this limitation [20,32]. Soft tissue thickness was higher in CTRL compared to AN patients. Previously published results on soft tissue thickness showed similar results compared to the present CTRL group [32]. Due to the novelty of the updated software, data on TBS_TT_ are limited. A posthoc analysis of postmenopausal women treated with denosumab in the FREEDOM trial showed a significant increase of TBS_TT_ and TBS_BMI_ from baseline, with a numerical greater increase of TBS_TT_ independently of BMD and an ongoing improvement of TBS_TT_ up to 10 years of denosumab treatment [20,33]. In the OsteoLaus study, TBS_TT_ was assessed in 1362 postmenopausal women with a high risk for fracture (BMI of 25.9±4.5 kg/m^2^) and followed for over more than 4 years. While TBS_TT_ correlated positively with BMI and body composition parameters, TBS_BMI_ was negatively correlated with BMI and body composition. However, both TBS algorithms showed similar performance as independent predictors for major osteoporotic fractures and TBS_TT_ was higher in individuals with higher BMI compared to TBS_BMI_, and mostly different in obese women [32]. In the present study TBS was applied in AN patients with low BMI, outlining the recommended BMI range by the manufacturer for clinical use. However, the primary potential impact of soft tissue outside the recommended BMI range is above a BMI of 37kg/m^2^. Both algorithms provided comparable results in AN patients and TBS was significantly lower compared to controls, which is in line with previous findings in adolescent females [30]. However, the already evident differences between TBS_TT_ and TBS_BMI_ in normal weight controls show the practical implication when comparing populations with differences in body composition.

The significantly reduced TBS in adult AN patients highlights a rarefication of trabecular network. A previous analysis of Watters et al. in adult AN patients showed in a subgroup of patients significantly reduced TBS_BMI_ and BMI was significantly lower in this subpopulation [34]. Assessment of bone microarchitecture in AN using HR-pQCT scanning, the most precise non-invasive in-vivo analysis, confirmed not only a significant reduced trabecular network in AN patients, but also a rarefication of cortical bone in the peripheral skeleton. Further, reduced finite element analysis-derived estimation of failure load indicated reduced bone strength in AN patients [28,29]. Since bone strength is determined in only 60–70% by BMD, the possibility to use TBS in clinical routine without additional radiation, makes it a suitable tool to estimate bone microarchitecture on top of BMD measurement.

We found a significant reduced BMD at all sites using DXA scanning, and therefore confirm previous reports in literature. A meta-analysis including over 21.600 patients with AN showed a reduction of BMD at all sites, lumbar spine, all femoral skeletal sites, radius and whole body assessment using DXA scans, compared to healthy controls. Compared to patients with bulimia nervosa, the prevalence of secondary osteoporosis in AN is much higher [24]. Further, disease duration and the presence of amenorrhea have been identified as key contributors for bone loss in AN patients [24,25]. In the present study, the most pronounced reduced BMD has been identified at the lumbar spine and a negative correlation with duration of secondary amenorrhea has been demonstrated. 73% of patients had secondary amenorrhea and these results are in line with previous reports in literature [26,27]. The risk factors for bone loss are multifactorial in AN patients. Laboratory assessment not only highlighted a disturbance of sexual hormonal axes, but also an uncoupling of bone turnover with increased bone resorption and reduced bone formation [35,36]. Further, serum sclerostin levels were increased in adult AN patients. Data on sclerostin in the literature are scarce and conflicting–while Maimon et al reported increased levels in young adult patients with AN (aged 18.4 ± 2.2years) compared to controls [6], Faje et al. found no difference in sclerostin levels in adolescents (aged 16.7 ± 0. 22years) [37]. Since sclerostin is increasing with age [38], these conflicting results can be interpreted in terms of an age effect, even more since the control group in Faje et al. was significantly younger compared to the AN patients.

Body composition analysis showed not only significantly reduced fat mass, but also reduced LBM and BMC compared to healthy controls. According to previously published normative data for body composition analysis using GE-Healthcare DXA systems, healthy controls were within normal range in all compartments and AN patients far below all cut-offs [23,39,40]. Lean body mass not only indicates muscle mass, but is also associated with muscular strength and predictive for health outcomes [41,42]. Percentage of LBM in AN patients was higher compared to controls and if compared to normative reference levels [39,40]. A compensatory mechanism for many AN patients is to increase physical activity to reduce body weight. LBMI measures total skeletal muscle mass scaled to height, allowing thereby a comparison of LBM in individuals with different height without being confounded by fat mass. In the present study population, not only a significant difference in weight and fat mass was observed, but healthy individuals of the CTRL group were significantly taller. Therefore, making LBMI the more reliable corrected parameter and confirming a significantly reduced LBM in AN.

This study has strengths and limitations. The study demonstrates not only lower BMD, but also lower trabecular bone texture parameters assessed by both available TBS algorithms in adult AN patients. TBS software was used outside the recommended BMI range for clinical use. However, the impact of soft tissue is crucial in patients outlining the upper reference level rather than the lower one. Both algorithms performed comparable in this patient population with very low soft tissue thickness. Further, TBS_TT_ covers a large range of tissue thickness over the region of interest and subsequently a larger rage of BMI. However, even in controls with normal body composition, the differences in soft tissue thickness have impact on texture assessment, which is especially important when comparing populations with differences in body composition. No information on comedication or compliance regarding routine supplementation of calcium and vitamin D in AN patients and controls was available, since no detailed assessment has been performed at the timepoint of DXA scanning and the retrospective study design. Further, blood sampling was performed standardized after over-night fasting within a specific timeframe in AN patients and controls, but was not further controlled for e.g. physical activity, diet or medication use.

To conclude, adult AN patients have markedly lower BMD at all measured sites, disturbances in sexual hormonal axes as well as an uncoupling of bone turnover. Bone texture analysis showed significantly lower TBS results compared to healthy CTRL, indicating impaired trabecular bone microarchitecture. No difference between the two TBS algorithms has been observed in AN patients since both algorithms account precisely the low soft tissue thickness as well as the low BMI in this cohort. However, within the CTRL group a difference between TBS_TT_ and TBS_BMI_ has been observed despite normal body composition. Therefore, TBS_TT_ seems to be a promising new tool to improve comparison of different cohorts especially with increasing soft tissue thickness.
